# Stable expression of aquaporins and hypoxia-responsive genes in adventitious roots are linked to maintaining hydraulic conductance in tobacco (*Nicotiana tabacum*) exposed to root hypoxia

**DOI:** 10.1371/journal.pone.0212059

**Published:** 2019-02-07

**Authors:** Xiangfeng Tan, Janusz J. Zwiazek

**Affiliations:** Department of Renewable Resources, University of Alberta, Edmonton, Alberta, Canada; Qingdao Agricultural University, CHINA

## Abstract

Formation of adventitious roots in plants is a common response to hypoxia caused by flooding. In tobacco, after one week of root hypoxia treatment, plants produced twice as many adventitious roots as the aerated plants, but their maximum length was reduced. Hypoxia severely reduced net photosynthesis, transpiration rates, and photosynthetic light responses. Relative transcript abundance of the examined aquaporins in lateral roots was reduced by hypoxia, but in adventitious roots it remained unchanged. This apparent lack of an effect of root hypoxia on the aquaporin expression likely contributed to maintenance of high hydraulic conductance in adventitious roots. Lateral roots had lower porosity compared with adventitious roots and the expression of the *ACS* (*1-aminocyclopropane-1-carboxylate synthase*) gene was induced in hypoxic lateral roots, but not in adventitious roots, providing additional evidence that lateral roots were more affected by hypoxia compared with adventitious roots. ATP concentrations were markedly lower in both hypoxic lateral and adventitious roots compared with aerated roots, while the expression of fermentation-related genes, *ADH1* (*alcohol dehydrogenase 1*) and *PDC1* (*pyruvate decarboxylase 1*), was higher in lateral roots compared with adventitious roots. Since root porosity was greater in adventitious compared with lateral roots, the results suggest that the improved O_2_ delivery and stable root aquaporin expression in adventitious roots were likely the key factors helping flooded tobacco plants maintain high rates of root hydraulic conductance and, consequently, shoot gas exchange.

## Introduction

Frequency of floods is predicted to increase globally due to the climate changes [1]. As O_2_ diffusion rate is extremely low in water compared to air, prolonged flooding can lead to O_2_ deprivation and reductions of growth and survival in terrestrial plant species [2]. Root O_2_ deficiency (hypoxia) limits respirational ATP synthesis and results in an energy crisis and toxicity due to a transition to glycolysis and fermentation [3]. Tobacco plants are susceptible to injury from flooding [4,5] with chlorosis and inhibition of leaf expansion reported in plants exposed for two days to waterlogging [6]. In field-grown tobacco, an inhibition of stem growth and a decrease of water potential occurred within six days following a flooding event [7].

Faced with hypoxia, plants need to reprogram transcription [8], and curtail energy-consuming processes such as DNA and protein synthesis and cell division [2]. Numerous hypoxia-responsive proteins and genes that have been identified by the application of proteomic approach and microarrays are associated with sugar metabolism, glycolysis, fermentation and hormonal regulation [9–12]. Alcohol dehydrogenase (ADH) and pyruvate decarboxylase (PDC) are the two key enzymes in fermentation and are commonly induced by hypoxia and anoxia in plants and fungi [13–15]. High activities of ADH and PDC are associated with improved survival of plants under low O_2_ conditions, mainly due to their involvement in alleviating energy crisis. [3]. However, the increase in fermentation may also lead to the accumulation of toxic end products, such as ethanol and acetaldehyde [8]. Genes encoding 1-Aminocyclopropane-1-Carboxylate (ACC) synthase (ACS) are also induced under O_2_ deficiency [16,17]. ACS catalyzes a regulatory step in endogenous ethylene synthesis, which is a major biochemical process in response to hypoxia [18].

Hydraulic adjustments are among the early responses of plants to flooding [19]. This is often manifested as wilting due to the loss of balance between water loss and uptake [20,21]. Aquaporins, including plasma membrane intrinsic proteins (PIPs), tonoplast intrinsic proteins, nodulin26-like intrinsic proteins, small basic intrinsic proteins, and the uncategorized intrinsic proteins, are membrane intrinsic proteins that can rapidly respond to various abiotic and biotic stresses including O_2_ deprivation [19,22]. The inhibition of aquaporin gating and root hydraulic conductivity in flooded plants may be regulated by cellular acidosis caused by the shift from respiration to fermentation [23], and depletion of ATP required for the phosphorylation of some aquaporins [20]. X-ray structure confirmed that the protonation of a conserved histidine residue under low pH during flooding resulted in the closed conformation of aquaporins [24]. In addition to regulating plant hydraulics, aquaporins are involved in the transport of other small molecules including CO_2_ [25,26], NH_3_ [27] and O_2_ [28]. Under flooding, transcription of *Arabidopsis* aquaporin NIP2;1 was induced [29], and shown to be a transporter of lactic acid that is produced by fermentation [30].

Plants vary in their flooding tolerance and adopt different survival strategies. These strategies include fast shoot elongation to escape from submergence [31] and development of suberized barriers in roots to reduce radial O_2_ loss [32,33]. Adventitious roots (ARs), are often induced in flooded plants. They may be placed close to the surface in some plants and usually have low gas diffusive resistance [34–36]. Six days of root hypoxia induced about twice as many ARs compared with aerated tobacco plants [37]. ARs promote internal diffusion of O_2_ from shoots to roots and elevate respiratory ATP production in roots [38]. Different types of biotic and abiotic stresses other than flooding can induce AR formation including wounding [39] and exogenous hormonal treatments [40]. Ethylene and other phytohormones have been implicated in the regulation of AR formation [21,37,41]. Different hormones may interact with each other in AR formation, but it still remains elusive how plants manage this complex regulation network in AR formation [42].

Formation of ARs has been highlighted as one of the most important adaptive traits under flooding in numerous species [43]. However, more evidence is still needed to evaluate the functional traits of ARs contributing to hypoxia tolerance. It was previously demonstrated that adventitious roots had higher hydraulic conductivity than similarly-sized lateral roots in flooded tamarack (*Larix laricina*) [44]. However, it remains unclear whether the high hydraulic conductivity of adventitious root is associated with aquaporin activities. In the present study, hydroponically grown tobacco plants were subjected to root hypoxia to shed more light on the processes in hypoxia-induced ARs that facilitate water transport. It was hypothesized that root hydraulics and ATP production under hypoxia can be enhanced by the formation of ARs. Root porosity, ATP contents and transcript profiles of hypoxia-responsive genes were compared between ARs and existing LRs. Transcription profiling of *PIP*s in ARs was also compared to LRs to examine potential significance of various PIPs in the responses of roots to hypoxia.

## Materials and methods

### Growth conditions and hypoxia treatment

Tobacco seeds were germinated and seedlings were grown in horticultural soil a controlled growth room with 18 h photoperiod, 22/18°C (day/night) temperature, 400 μmol m^-2^ s^-1^ photosynthetic photon flux density, 350 μmol mol^−1^ CO_2_ and 50% relative humidity. After 3 weeks of growth, plants were transferred to 40-L plastic tubs (~ 60×40×20 cm) containing 50% strength modified aerated Hoagland’s solution [45]. Thirty-two plants were randomly selected and grown in four tubs (8 plants in each tub). After one week, 16 plants in two tubs were subjected to hypoxia by flushing nitrogen gas (99.998%, Praxair, Danbury, CT, USA) through the solution to reach a dissolved O_2_ level of ~ 2 mg L^-1^ and then leaving the solution stagnant. The other 16 plants in two tubs were well-aerated with air pumps and served as control (dissolved O_2_ concentration of ~ 8 mg L^-1^).

### Gas exchange and photosynthetic light responses

After two days and one week of treatments, gas exchange was measured with the Li-Cor LI-6400XT portable photosynthesis system equipped with the 2×3 cm^2^ red-blue light chamber (Li-Cor, Lincoln, NE, USA). Six plants in each treatment and three middle-position fully expanded leaves on each plant were randomly selected. Net photosynthetic rate (*P*_n_), transpiration (*E*) and stomatal conductance (*g*_s_) were measured. Air flow rate was set to 400 μmol s^-1^, photosynthetic photon flux density (PPFD) was 400 μmol m^-2^ s^-1^, and reference CO_2_ concentration was 400 μmol mol^-1^. An automated program of LI-6400XT was used to determine photosynthetic light responses starting at PPFD of 1500 μmol m^-2^ s^-1^, followed by 1200, 1000, 800, 500, 300, 200, 100, 50, 20 and 0 μmol m^-2^ s^-1^. Three plants in each treatment were randomly selected and net photosynthesis was auto-logged when it reached a steady rate. A modified rectangular hyperbole model was employed to estimate light saturated *P*_n_ (*P*_m_), light saturation point (I_m_) and light compensation point (I_c_) [46].

### Root hydraulic conductance (K_*r*_)

A high–pressure flow meter (HPFM, Dynamax Incorporated, Houston, TX, USA) was used to measure tobacco root *K*_r_ as previously described [47,48]. Shoots were excised about 2 cm above the root collar, and the roots were connected to the HPFM. Roots were kept in treatment solutions during the measurements. Water was forced into roots at increasing pressures (0 to 0.5 MPa) and linear regression between applied pressure and flow rate was used to obtain a slope of the relationship which represented *K*_r_.

### Number, dry mass and maximum length of ARs

After one week of treatment, number of ARs was counted and the maximum length of ARs was measured with a ruler. Dry mass of ARs was determined after drying the roots in an oven at 85°C.

### Root porosity

Root porosity in lateral (LR) and adventitious (AR) roots was estimated with the pycnometer method based on Archimedes’ principle [49] after one week of treatment using the following equation:

$$Porosity\;\left(\%\right)=100\times \left(M_{h}‑M_{r+w}\right)/\left(M_{w}+M_{r}‑M_{r+w}\right)$$

where M_w_ is mass of the water filled pycnometer, M_r_ is mass of roots, M_r+w_ is mass of pycnometer with roots and water and M_h_ is mass of pycnometer with homogenized roots [49].

### Root ATP concentration

LRs and ARs were sampled and ground in liquid nitrogen after one week of hypoxia treatment. ATP concentration was determined with the ENLITEN ATP Assay Kit (Promega, Madison, WI, USA) by measuring bioluminescence and quantified with a standard curve using ATP standard (Promega). Luminescence signal was detected using a microplate reader (Fluostar Optima, BMG Labtech, Ortenberg, Germany) as previously described [28].

### RNA transcription profiling

After two days and one week of treatment, LRs and ARs from 6 plants in each treatment were sampled. The samples were quickly frozen and kept in liquid nitrogen before being transferred to the –80°C freezer. The samples were ground with a mortar and pestle in liquid nitrogen. Total RNA was extracted with a Plant RNeasy extraction kit (Qiagen, Valencia, CA USA). First strand of cDNA was synthesized from 1μg total RNA using a Reverse Transcription Kit (Qiagen). Quantitative RT-PCR was employed to analyze relative RNA expression using the 2^–ΔCt^ method. The relative transcript abundance of *PIP*s was normalized against geometric mean of the CT value of two reference genes, *NtEF1-α* (AF120093) and *L25* (L18908) [50]. Transcripts levels of *NtPIP1;1* (AF440271), *PIP1;2* (= *AQP1*, AF024511), *PIP1;3* (U62280), *PIP1;4* (DQ914525) and *PIP2;1* (AF440272) were analyzed. These aquaporins were selected because of their sequence availability in the public database and earlier studies showing their functional importance [25,28]. *NtADH1* (*alcohol dehydrogenase 1*, X81853.1) and *PDC1* (*pyruvate decarboxylase 1*, X81854.1) were selected as hypoxic indicators. Relative transcript abundance of *ACS* (X65982.1) was also determined since it encodes an enzyme catalyzing a rate-limiting step in ethylene synthesis [51]. Gene-specific primers are described in S1 Table.

### Statistical analysis

Means (*n* = 3–6) and standard errors (SE) were calculated. Paired t-test was performed to compare AR formation and *K*_r_ between aerated and hypoxic roots (α = 0.05). In all other comparisons, one-way ANOVA followed by Tukey’s test was performed to compare means (α = 0.05). Three out of four replications of *PDC1* relative transcript abundance of aerated LRs were too low to be detected and consequently only the relative transcript abundance of *PDC1* of aerated ARs, hypoxic LRs and hypoxic ARs were compared in Tukey’s test.

## Results

### Leaf gas exchange and photosynthetic light responses

**Fig 1:**
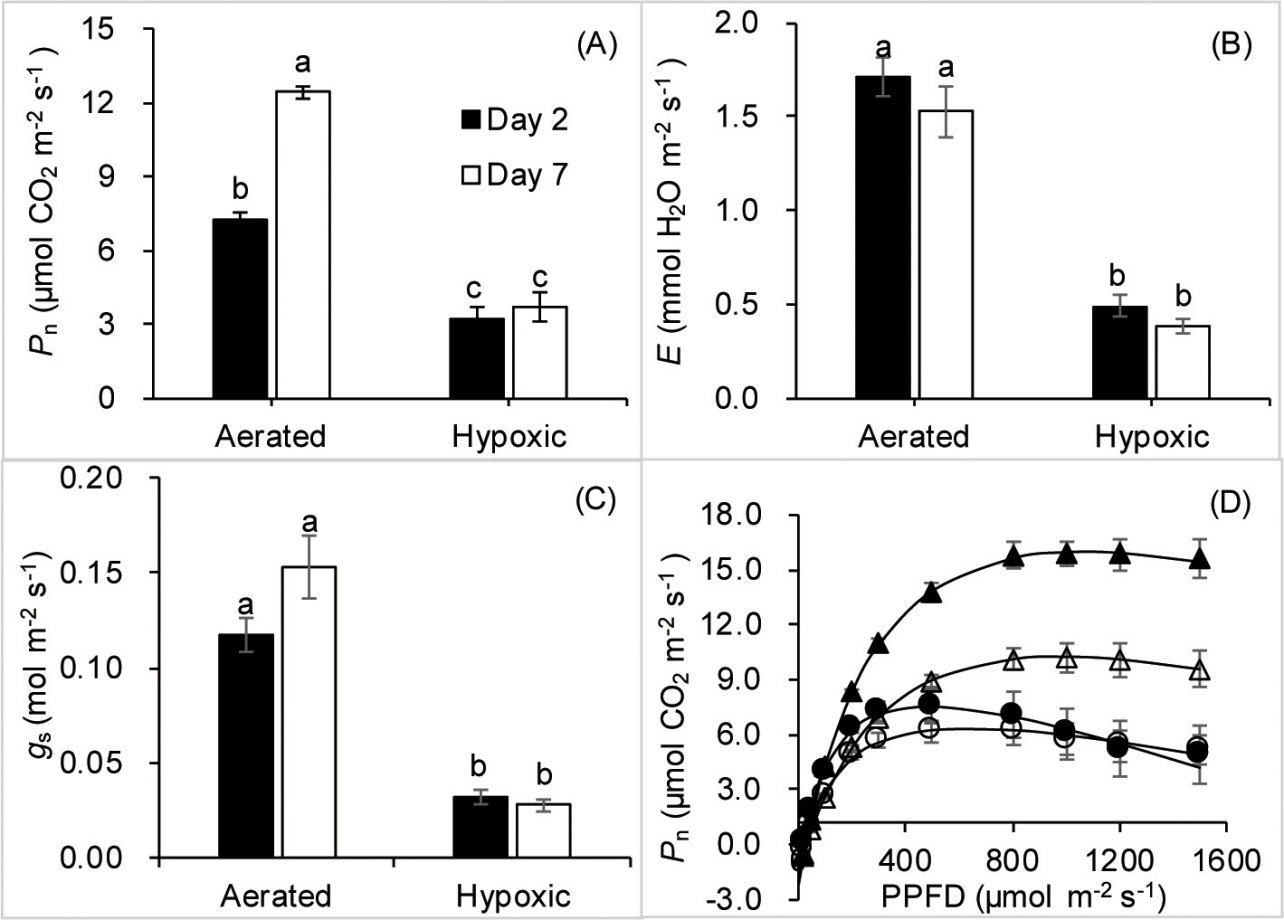
**Net photosynthesis (*P***_**n**_**, A), transpiration rates (*E*, B), stomatal conductance (*g***_**s**_**, C) and photosynthetic light responses (D) of well-aerated tobacco plants and plants subjected to root hypoxia treatment for two days and one week.** Means ± SE (*n* = 5 or 6 for *P*_n_, *E* and *g*_s_. and *n* = 3 for photosynthetic light responses) are shown. Different letters above the bars indicate statistically significant differences determined by the Tukey’s test after one-way ANOVA (*P* ≤ 0.05). Aerated after two days (open triangles), hypoxia after two days (open circles), aerated after one week (black triangles), and hypoxia after one week (black circles).

After one week of treatment, the leaves of hypoxic plants showed no signs of chlorosis or wilting. *P*_n_ of hypoxic plants decreased by over 50% and 70% compared with aerated plants after two days and one week of treatment, respectively (Fig 1A). Hypoxic plants also showed a decrease of more than 70% in *E* and *g*_s_ on the two treatment days (Fig 1B and 1C).

Hypoxia treatment profoundly affected photosynthetic light responses (Fig 1D). Both two days and one week of hypoxia treatments resulted in significant declines of I_c_ and I_m_ (Table 1). *P*_m_ of hypoxic plants also showed a significant decrease compared with aerated plants after one week of treatment (Table 1).

**Table 1:** Comparison of estimated light-saturated photosynthesis (*P*_m_, μmol CO_2_ m^-2^ s^-1^), light saturation point (I_m_, μmol m^-2^ s^-1^) and light compensation point (I_c_, μmol m^-2^ s^-1^) in photosynthetic light responses of tobacco plants subjected to aeration and hypoxia treatment for two days and one week.

	Aerated DAY2	Hypoxia DAY2	Aerated DAY7	Hypoxia DAY7
*P*_m_	11.47 ± 0.82 a	7.39 ± 0.79 a	17.85 ± 0.87 b	8.76 ± 1.2 a
I_m_	1045.99 ± 84.98 a	683.54 ± 40.39 b	1129.38 ± 52.12 a	551.66 ± 103.49 b
I_c_	28.69 ± 2.55 a	19.7 ± 0.11 b	26.34 ± 1.94 a	14.524 ± 3.68 b

Means ± SE (*n* = 3) are shown. Different letters indicate statistically significant differences determined by the Tukey’s test after one-way ANOVA (*P* ≤ 0.05)

### AR formation and root porosity

After one week of treatment, root mortality was observed in the lower part of hypoxic roots. Formation of ARs was induced by hypoxia around the stem base (Table 2). Hypoxic plants had over two-fold higher AR number compared with aerated plants. However, the maximum length of ARs in hypoxic plants was significantly lower compared with aerated plants (Table 2). Hypoxia treatment did not affect the total dry mass of ARs (Table 2).

**Table 2:** Number, maximum length, and dry mass of adventitious roots (ARs) in aerated and hypoxia treatment for one week.

	ARs	
	Aerated	Hypoxic
Number	14 ± 1.51	28.17 ± 2.27*
Maximum length	5.77 ± 0.46*	3.53 ± 0.18
Dry mass	0.059 ± 0.012	0.038 ± 0.007

Means ± SE are shown (*n* = 6 for number and maximum length, and *n* = 8 for dry mass of ARs). Asterisks indicate significance between aerated and hypoxic ARs determined by the t-test (*P* ≤ 0.05).

**Fig 2:**
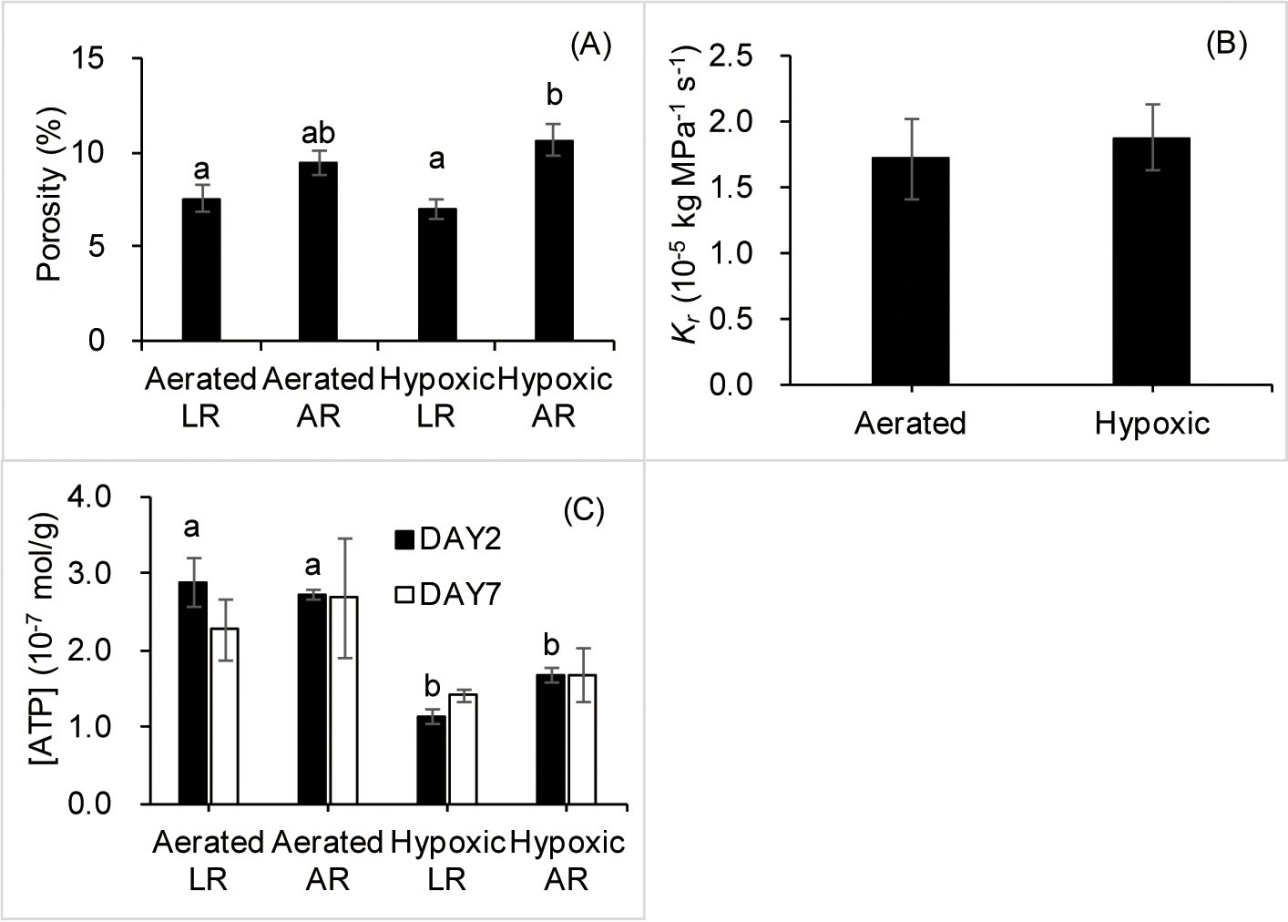
**Root porosity of adventitious (ARs) and lateral roots (LRs) (A) and hydraulic conductance (*K***_**r**_**, B) of tobacco plants subjected to aerated or root oxygen deprivation (hypoxia) treatments for one week, and ATP concentration of ARs and LRs (C) after two days and one week of treatment.** Means ± SE (*n* = 4–6 for porosity test, *n* = 6 for *K*_*r*_ and *n* = 3 for ATP assay) are shown. Different letters above the bars indicate statistically significant differences determined by the Tukey’s test after one-way ANOVA (*P* ≤ 0.05).

Root air space was estimated by the root porosity test after one week of treatment. Hypoxia lead to a 13% increase of porosity in ARs and a slight decrease of porosity in LRs (Fig 2A). The porosity of hypoxic ARs was higher by over 50% compared with hypoxic LRs. The porosity of aerated ARs was higher by over 25% compared with aerated LRs.

### K_*r*_ and root ATP concentration

There was no significant difference in *K*_r_ between hypoxic roots and aerated roots after one week of treatment (Fig 2B).

After two days of treatment, hypoxia resulted in a significant decrease of ATP concentration in both ARs and LRs (Fig 2C). ATP concentration of hypoxic LRs and ARs decreased by about 60% and 40%, respectively, compared with aerated roots (Fig 2C). After one week of treatment, no significant difference in ATP concentration between hypoxic and aerated roots was detected.

### RNA expression profiling in roots

**Fig 3:**
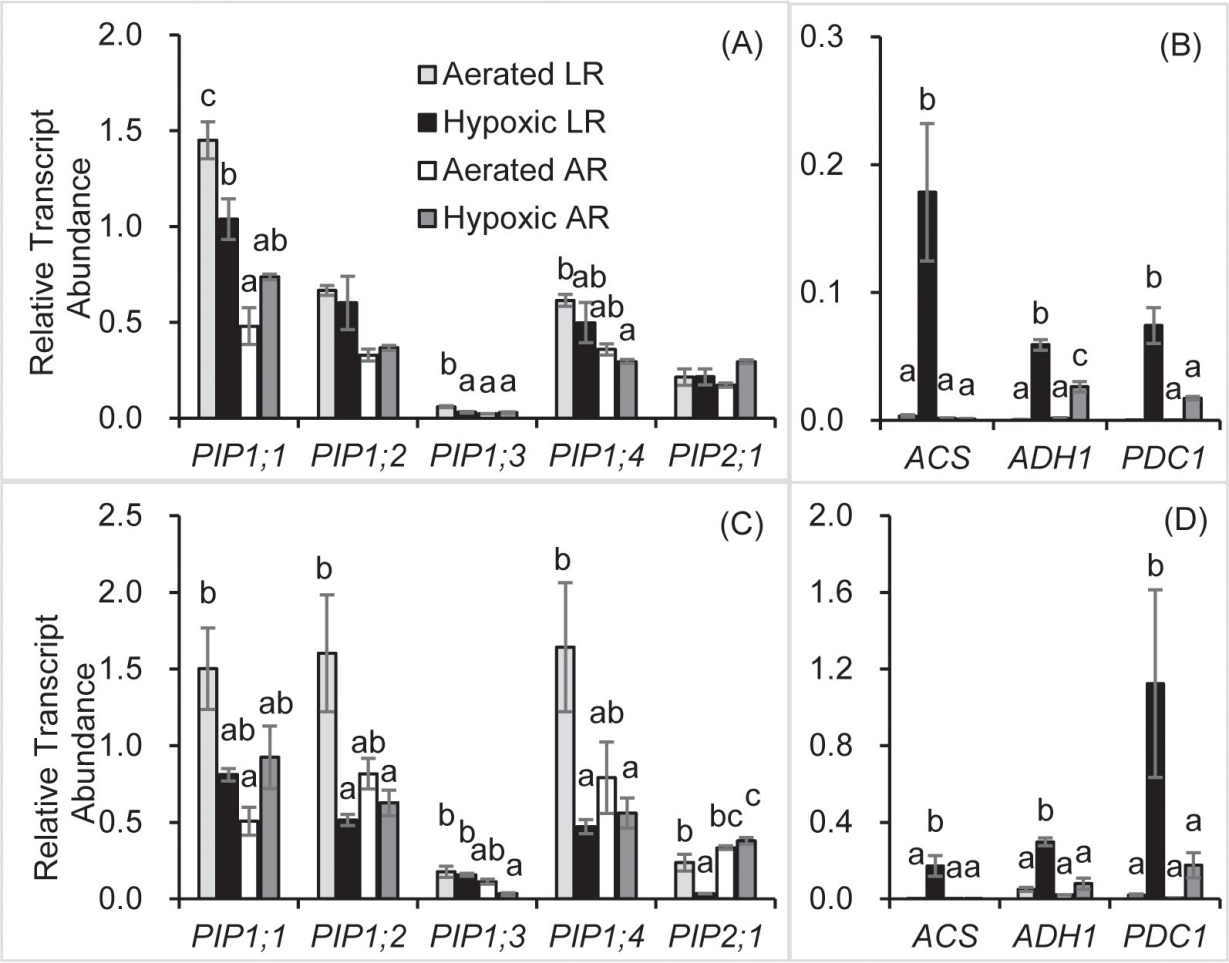
**Relative transcript abundance of well-aerated tobacco plants and plants subjected to root hypoxia treatment for two days (A and B) and one week (C and D).** Relative transcript abundance of plasma membrane intrinsic proteins (*PIP*s, A and C), *1-aminocyclopropane-1-carboxylate synthase* (*ACS*), *alcohol dehydrogenase 1* (*ADH1*) and *pyruvate decarboxylase 1* (*PDC1*) (B and D) is shown. Means ± SE are shown (*n* = 4–6). *PDC1* expression of LRs aerated after two days is not shown because of un-determined values. Different letters above the bars indicate statistically significant differences determined by the Tukey’s test after one-way ANOVA (*P* ≤ 0.05).

Hypoxia resulted in significant decreases of *PIP1;1* and *PIP1;3* relative transcript abundance in LRs after two days of treatment (Fig 3A). However, relative transcript abundance in ARs remained unchanged (Fig 3A). Relative transcript abundance of *ACS*, *ADH1* and *PDC1* in LRs were sharply induced by hypoxia (Fig 3B). Hypoxic ARs also showed significantly higher *ADH1* relative transcript abundance compared with aerated ARs, however, *ACS* remained unchanged (Fig 3B).

After one week of treatment, relative transcript abundance of *PIP1;2*, *PIP1;4* and *PIP2;1* in hypoxic LRs were significantly lower compared with the aerated LRs, whereas relative transcript abundance of *PIP1;1* and *PIP1;3* showed no change (Fig 3C). In contrast, hypoxia did not result in significant changes of *PIP* relative transcript abundance in ARs (Fig 3C). Relative transcript abundance of *ACS*, *ADH1* and *PDC1* exhibited similar trends as on day two (Fig 3D). A sharp increase of *ACS*, *ADH1* and *PDC1* expression was triggered in LRs by hypoxia (Fig 3D). Hypoxic ARs showed no changes in relative transcript abundance of the *ACS*, *ADH1* and *PDC1* compared with aerated ARs (Fig 3D).

## Discussion

O_2_ deficiency is a challenging environmental factor that produces complex responses in plants. Following two days and one week of treatment, gas exchange in tobacco was sharply reduced by hypoxia. Hypoxia also increased the number of ARs but with a similar biomass as in aerated plants. ARs showed different response patterns to hypoxia compared with the LRs in terms of the transcript profiles of *PIP*s and hypoxia-responsive genes, which may partially contribute to maintaining *K*_r_ of hydroponically-grown tobacco plants.

Stomatal conductance, which is regulated by hydraulic and (or) chemical signals, is the main limiting factor for leaf carbon assimilation [52]. The decline in *P*_n_ after two days and one week of hypoxia was likely due to the stomatal closure, which was reflected by the decreased *E*. The decrease in stomatal opening, as demonstrated by lower *g*_s_, was likely responsible for the reductions in photosynthetic light responses, which showed that *P*_n_ of hypoxic plants was saturated at a significantly lower light intensity. Reduced *g*_s_ of hypoxic leaves limits internal CO_2_ concentration used for carbon assimilation. Root hypoxia also reduced I_c_, the light intensity at which the rate of total photosynthesis is balanced by the rate of respiration suggesting that root hypoxia inhibited leaf respiration in addition to photosynthesis. However, hypoxic plants maintained positive *P*_n_ at similar rates on days two and seven, which demonstrates relative tolerance of tobacco plants of hypoxic conditions.

One week of root hypoxia did not influence *K*_r_ in hydroponically grown tobacco. Effects of O_2_ deprivation on root hydraulics varies between plant species and experimental conditions. While some studies reported reduced *K*_r_ in response to O_2_ deficiency [53,54], other studies showed no effect [44,55]. In contrast to *K*_*r*_, hypoxia resulted in a significant decrease of *E* in the present study. *E* and *K*_*r*_ are frequently strongly linked in plants [56], but this relationship may also be affected by other factors including leaf to root ratio [57]. Root growth is typically reduced more than leaf growth under hypoxic conditions [58]. In the present study, leaves of hypoxic plants showed no chlorosis and wilting, but root mortality was observed. Thus, even though *K*_r_ remained unchanged under hypoxic conditions, the increasing transpiration demand, as a result of leaf growth, may still lead to decreased *E* in the present study. The diurnal changes of root hydraulic conductivity were also found to be independent of transpiration in flooded *Zea mays* [59]. More likely, the signal triggering hydraulic adjustment either does not originate in the stomata or is impaired by the secondary changes caused by hypoxia.

It appears that the number of ARs, not its overall length and dry mass, was the overriding factor in maintaining root *K*_r_ in hypoxic tobacco plants. Even though root porosity was similar in aerated and hypoxia treatments, the number of ARs was increased by hypoxia. The formation of ARs is an important adaptation to low O_2_ conditions in some plants [2,34]. In the present study, hypoxia induced the formation of over twice as many ARs compared with aerated tobacco plants, but their length was reduced by hypoxia. Similar results were previously reported in tobacco and the authors concluded that the formation of short ARs cannot functionally replace the primary root system which contributed to the relative intolerance of tobacco to O_2_ deficiency [37]. However, in the present study, *K*_r_ in hypoxic plants showed no change compared with aerated plants. Additionally, *P*_n_ and *E* of hypoxic plants showed no further decrease after one week of hypoxia compared with two days of treatment. These results indicate that the ARs were effective in maintaining root water transport. The roles of hypoxia-induced ARs are not limited to replacing existing roots. ARs induced by hypoxia had higher porosity than the LRs and could conduct more air from shoots to roots and the rhizosphere (radial O_2_ loss, ROL). ROL of ARs may have a profound influence on rhizosphere aeration and nutrient availability in plants under O_2_ deficiency [60]. Thus, the numerous short ARs induced by hypoxia in this study could potentially affect tobacco rhizosphere aeration and contribute to the survival and functioning of the root system under hypoxia.

Decreased root hydraulic conductivity of anoxic *Arabidopsis* has been linked to aquaporin closure [23], and a structure-based protonation mechanism under O_2_ deprivation conditions has been demonstrated [23,24]. In addition to aquaporin gating analysis, aquaporin gene expression patterns can reveal the importance of various aquaporins in plant responses to O_2_ deficiency [19]. Although transcripts may not be translated under certain conditions and posttranslational regulation can modify the function of aquaporins, transcriptional responses can shed light on the translational potential of cells experiencing O_2_ deficiency [61]. Microarray analysis showed that aquaporin expression was downregulated in O_2_ deficient *Arabidopsis* [29] and *Persea americana* [62]. Here, the relative transcript abundance of *PIP*s in ARs was compared with LRs. After two days of hypoxia, relative transcript abundance of *PIP1;1* and *PIP1;3* decreased in LRs but showed no change in ARs. One week of hypoxia resulted in decreased relative transcript abundance of *PIP*s in LRs except *PIP1;1* and *PIP1;3*, while hypoxic ARs showed unchanged expression of *PIP*s on day seven. These results indicate that the relative transcript abundance of *PIP*s in ARs was less affected by hypoxia, which may contribute to hydraulic adjustment. In addition to regulating water transport, PIPs impact other physiological processes. Tobacco PIP1;2 has been reported to be permeable to CO_2_ [25]. CO_2_ can accumulate in roots of plants growing in stagnant water due to its low diffusion rate in water and high concentrations of CO_2_ can cause cell acidification [60]. Thus, the down-regulation of *PIP1;2* may lead to decreased efflux of CO_2_ in cells and intensify cell acidification. Interestingly, expression of *PIP1;1* and *PIP1;3* in LRs showed different responsive patterns compared with the other examined *PIP* genes under hypoxia. Further research is needed to shed more light on the biological roles of PIP1;1 and PIP1;3 in tobacco. Tobacco PIP1;3 has been shown to be potentially involved in the O_2_ transmembrane transport [28]. Although the experimental set-up of the two experiments are similar, the objective of this study was mainly comparing the differences between ARs and LRs under hypoxic conditions. However, unlike the present study, it was reported that the relative transcript abundance of tobacco *PIP1;3* was upregulated by root hypoxia [28]. The reason for the difference may be that in the present study LRs and newly formed ARs were sampled separately rather than the whole root. Additionally, gene expression patterns in response to abiotic stresses may vary between different developmental stages [63]. Tobacco plants were exposed to root hypoxia three weeks after germination in the present study while plants were subjected to root hypoxia two weeks after germination in the study of Zwiazek *et al*. [28], which may also contribute to the differences in results. Since ARs were less affected by hypoxia than LRs in terms of *PIP* transcription, ARs can likely more actively participate in root water uptake under hypoxic conditions. In fact, ARs induced by hypoxia in *Larix laricina* were shown to have higher hydraulic conductivity than the existing roots [44]. However, it should be emphasized that both transcriptional and post-transcriptional regulation may affect aquaporin abundance and functions and several studies have shown the lack of correlation between aquaporin mRNA and protein abundance [64–66]. It remains to be determined whether the changes in transcript abundance observed in the present study are functionally significant.

Phytohormones, especially ethylene, are involved in the regulation network in response to O_2_ deficiency, including the initiation and regulation of ARs [67,68]. In this study, transcript profiling showed that more *ACS* transcripts were induced in hypoxic LRs than ARs, which could result in the accumulation of ACC in hypoxic LRs. ACC, an ethylene synthesis precursor, is induced in flooded roots while the conversion of ACC to ethylene needs the presence of O_2_ [69]. The transport of ACC from stressed roots to other tissues serves as a signal and causes the formation of ARs in aerated shoots [51]. Both *ADH* and *PDC* were frequently reported to be up-regulated in O_2_ deficient tissues [13,62]. In this study, transcripts of tobacco *ADH1* and *PDC1* were sharply up-regulated in hypoxic LRs, while ARs showed much lower expression. Despite the inefficiency of ethanolic fermentation, the activities of ADH and PDC are essential to plants under low O_2_ conditions to meet the energy demand [3]. However, the accumulation of end products of fermentation is toxic [3]. In the present study, two days of hypoxia resulted in a significant decline of ATP concentrations in both ARs and LRs. Although hypoxic ARs did not differ from hypoxic LRs in ATP concentration, hypoxic ARs maintained markedly lower levels of *ADH* and *PDC* transcripts compared with LRs. This suggests that the better aeration of ARs reduced their dependence on inefficient and toxic fermentation compared with LRs under low O_2_ conditions.

In conclusion, the formation of short ARs was induced by hypoxia in hydroponically grown tobacco plants. Although, the length of ARs was reduced in hypoxic plants, it appears that they were likely more effective in facilitating O_2_ diffusion into the roots and maintaining low level of fermentation and high *K*_r_. *PIP* expression patterns likely reflect the metabolic status in roots and may also be related to gas transport. Stable *PIP* transcripts of ARs under hypoxic conditions may be indicative of an active role of ARs in root water transport under hypoxic conditions.

## Supporting information

S1 Dataset Parameters recorded in this study. (XLSX)

S1 Table qRT-PCR primers for tobacco (*Nicotiana tabacum*) genes. (DOCX)

## Abbreviations

ACC: 1-aminocyclopropane-1-carboxylate
ACS: 1-aminocyclopropane-1-carboxylate synthase
ADH: alcohol dehydrogenase
AR: adventitious root
*g*_s_: stomatal conductance
HPFM: high–pressure flow meter
I_c_: light compensation point
I_m_: light saturation point
*K*_r_: root hydraulic conductance
LR: lateral root
PDC: pyruvate decarboxylase
PIP: plasma membrane intrinsic protein
*P*_m_: light saturated net photosynthesis
*P*_n_: net photosynthetic rate
PPFD: photosynthetic photon flux density
ROL: radial oxygen loss
*T*_r_: transpiration rate

## References

[1] Hirabayashi Y, Mahendran R, Koirala S, Konoshima L, Yamazaki D, Watanabe S, et al Global flood risk under climate change. Nat Clim Change. 2013;3: 816–821.

[2] Bailey-Serres J, Voesenek LACJ. Flooding stress: acclimations and genetic diversity. Annu Rev Plant Biol. 2008;59: 313–339. 10.1146/annurev.arplant.59.032607.092752 18444902

[3] Drew MC. Oxygen deficiency and root metabolism: injury and acclimation under hypoxia and anoxia. Annu Rev Plant Physiol Plant Mol Biol. 1997;48: 223–250. 10.1146/annurev.arplant.48.1.223 15012263

[4] Kramer PJ, Jackson WT. Causes of injury to flooded tobacco plants. Plant Physiol. 1954;29: 241–245. 16654650 10.1104/pp.29.3.241 PMC540505

[5] Padakandla SR. Climate sensitivity of crop yields in the former state of Andhra Pradesh, India. Ecol Indic. 2016;70: 431–438.

[6] Yu Q, Rengel Z. Waterlogging influences plant growth and activities of superoxide dismutases in narrow-leafed lupin and transgenic tobacco plants. J Plant Physiol. 1999;155: 431–438.

[7] Hunt PG, Campbell RB, Sojka R, Parsons JE. Flooding-induced soil and plant ethylene field-grown tobacco. Plant Soil. 1981;439: 427–439.

[8] Gibbs J, Greenway H. Mechanisms of anoxia tolerance in plants. I. Growth, survival and anaerobic catabolism. Funct Plant Biol. 2003;30: 1–47. 10.1071/PP98095 32688990

[9] Sachs MM, Freeling M, Okimoto R. The anaerobic proteins of maize. Cell. 1980;20: 761–767. 7418006 10.1016/0092-8674(80)90322-0

[10] Chang WW, Huang L, Shen M, Webster C, Burlingame AL, Roberts JK. Patterns of protein synthesis and tolerance of anoxia in root tips of maize seedlings acclimated to a low-oxygen environment, and identification of proteins by mass spectrometry. Plant Physiol. 2000;122: 295–318. 10677424 10.1104/pp.122.2.295 PMC58868

[11] Lee SC, Mustroph A, Sasidharan R, Vashisht D, Pedersen O, Oosumi T, et al Molecular characterization of the submergence response of the *Arabidopsis thaliana* ecotype Columbia. New Phytol. 2011;190: 457–471. 10.1111/j.1469-8137.2010.03590.x 21231933

[12] Gibbs DJ, Lee SC, Isa NM, Gramuglia S, Fukao T, Bassel GW, et al Homeostatic response to hypoxia is regulated by the N-end rule pathway in plants. Nature. 2011;479: 415–418. 10.1038/nature10534 22020279 PMC3223408

[13] Mugnai S, Marras AM, Mancuso S. Effect of hypoxic acclimation on anoxia tolerance in *Vitis* roots: response of metabolic activity and K^+^ fluxes. Plant Cell Physiol. 2011;52: 1107–1116. 10.1093/pcp/pcr061 21551160

[14] Lee MO, Hwang JH, Lee DH, Hong CB. Gene expression profile for *Nicotiana tababum* in the early phase of flooding stress. J Plant Biol. 2007;50: 496–503.

[15] Mustroph A, Lee SC, Oosumi T, Zanetti ME, Yang H, Ma K, et al Cross-kingdom comparison of transcriptomic adjustments to low-oxygen stress highlights conserved and plant-specific responses. Plant Physiol. 2010;152: 1484–1500. 10.1104/pp.109.151845 20097791 PMC2832244

[16] Mekhedov SL, Kende H. Submergence enhances expression of a gene encoding 1-aminocyclopropane-1-carboxylate oxidase in deepwater rice. Plant Cell Physiol. 1996;37: 531–537. 8759917 10.1093/oxfordjournals.pcp.a028976

[17] Rieu I, Cristescu SM, Harren FJM, Huibers W, Voesenek LACJ, Mariani C, et al *RP-ACS1*, a flooding-induced 1-aminocyclopropane-1-carboxylate synthase gene of *Rumex palustris*, is involved in rhythmic ethylene production. J Exp Bot. 2005;56: 841–849. 10.1093/jxb/eri078 15642709

[18] Voesenek LACJ, Sasidharan R. Ethylene–and oxygen signalling–drive plant survival during flooding. Plant Biol. 2013;15: 426–435. 10.1111/plb.12014 23574304

[19] Tan X, Xu H, Khan S, Equiza MA, Lee SH, Vaziriyeganeh M, et al Plant water transport and aquaporins in oxygen-deprived environments. J Plant Physiol. 2018;227: 20–30. 10.1016/j.jplph.2018.05.003 29779706

[20] Kamaluddin M, Zwiazek JJ. Ethylene enhances water transport in hypoxic aspen. Plant Physiol. 2002;128: 962–969. 10.1104/pp.010791 11891251 PMC152208

[21] Islam MA, MacDonald SE, Zwiazek JJ. Responses of black spruce (*Picea mariana*) and tamarack (*Larix laricina*) to flooding and ethylene. Tree Physiol. 2003;23: 545–552. 12730046 10.1093/treephys/23.8.545

[22] Maurel C, Boursiac Y, Luu DT, Santoni V, Shahzad Z, Verdoucq L. Aquaporins in plants. Physiol Rev. 2015;95: 1321–1358. 10.1152/physrev.00008.2015 26336033

[23] Tournaire-Roux C, Sutka M, Javot H, Gout E, Gerbeau P, Luu DT, et al Cytosolic pH regulates root water transport during anoxic stress through gating of aquaporins. Nature. 2003;425: 393–397. 10.1038/nature01853 14508488

[24] Törnroth-Horsefield S, Wang Y, Hedfalk K, Johanson U, Karlsson M, Tajkhorshid E, et al Structural mechanism of plant aquaporin gating. Nature. 2006;439: 688–694. 10.1038/nature04316 16340961

[25] Uehlein N, Lovisolo C, Siefritz F, Kaldenhoff R. The tobacco aquaporin NtAQP1 is a membrane CO_2_ pore with physiological functions. Nature. 2003;425: 734–737. 10.1038/nature02027 14520414

[26] Navarro-Ródenas A, Xu H, Kemppainen M, Pardo AG, Zwiazek JJ. *Laccaria bicolor* aquaporin LbAQP1 is required for Hartig net development in trembling aspen (*Populus tremuloides*). Plant Cell Environ. 2015;38: 2475–2486. 10.1111/pce.12552 25857333

[27] Jahn TP, Møller ALB, Zeuthen T, Holm LM, Klærke DA, Mohsin B, et al Aquaporin homologues in plants and mammals transport ammonia. FEBS Lett. 2004;574: 31–36. 10.1016/j.febslet.2004.08.004 15358535

[28] Zwiazek JJ, Xu H, Tan X, Navarro-Ródenas A, Morte A. Significance of oxygen transport through aquaporins. Sci Rep. 2017;7: 40411 10.1038/srep40411 28079178 PMC5227684

[29] Liu F, Vantoai T, Moy LP, Bock G, Linford LD, Quackenbush J. Global transcription profiling reveals comprehensive insights into hypoxic response in *Arabidopsis*. Plant Physiol. 2005;137: 1115–1129. 10.1104/pp.104.055475 15734912 PMC1065411

[30] Choi W-G, Roberts DM. *Arabidopsis* NIP2;1, a major intrinsic protein transporter of lactic acid induced by anoxic stress. J Biol Chem. 2007;282: 24209–24218. 10.1074/jbc.M700982200 17584741

[31] Van der Sman A, Voesenek LACJ, Blom C, Harren F, Reuss J. The role of ethylene in shoot elongation with respect to survival and seed output of flooded *Rumex maritimus* L. plants. Funct Ecol. 1991;5: 304–313.

[32] De Simone O, Haase K, Müller E, Junk WJ, Hartmann K, Schreiber L, et al Apoplasmic barriers and oxygen transport properties of hypodermal cell walls in roots from four amazonian tree species. Plant Physiol. 2003;132: 206–217. 10.1104/pp.102.014902 12746526 PMC166966

[33] Abiko T, Kotula L, Shiono K, Malik AI, Colmer TD, Nakazono M. Enhanced formation of aerenchyma and induction of a barrier to radial oxygen loss in adventitious roots of *Zea nicaraguensis* contribute to its waterlogging tolerance as compared with maize (*Zea mays* ssp. mays). Plant Cell Environ. 2012;35: 1618–1630. 10.1111/j.1365-3040.2012.02513.x 22471697

[34] Calvo-Polanco M, Señorans J, Zwiazek JJ. Role of adventitious roots in water relations of tamarack (*Larix laricina*) seedlings exposed to flooding. BMC Plant Biol. 2012;12: 99 10.1186/1471-2229-12-99 22738296 PMC3431261

[35] Ayi Q, Zeng B, Liu J, Li S, van Bodegom PM, Cornelissen JHC. Oxygen absorption by adventitious roots promotes the survival of completely submerged terrestrial plants. Ann Bot. 2016;118: 675–683. 10.1093/aob/mcw051 PMC5055620 27063366

[36] Herzog M, Striker GG, Colmer TD, Pedersen O. Mechanisms of waterlogging tolerance in wheat—a review of root and shoot physiology. Plant Cell Environ. 2016;39: 1068–1086. 10.1111/pce.12676 26565998

[37] McDonald MP, Visser EJW. A study of the interaction between auxin and ethylene in wild type and transgenic ethylene-insensitive tobacco during adventitious root formation induced by stagnant root zone conditions. Plant Biol. 2003;5: 550–556.

[38] Bailey-Serres J. Flood adaptive traits and processes: an overview. New Phytol. 2015;206: 57–73. 10.1111/nph.13209 25580769

[39] Ahkami AH, Lischewski S, Haensch KT, Porfirova S, Hofmann J, Rolletschek H, et al Molecular physiology of adventitious root formation in *Petunia hybrida* cuttings: involvement of wound response and primary metabolism. New Phytol. 2009;181: 613–625. 10.1111/j.1469-8137.2008.02704.x 19076299

[40] Pagnussat GC, Simontacchi M, Puntarulo S, Lamattina L. Nitric oxide is required for root organogenesis. Plant Physiol. 2002;129: 954–956. 10.1104/pp.004036 12114551 PMC1540240

[41] Steffens B, Wang J, Sauter M. Interactions between ethylene, gibberellin and abscisic acid regulate emergence and growth rate of adventitious roots in deepwater rice. Planta. 2006;223: 604–612. 10.1007/s00425-005-0111-1 16160845

[42] Bellini C, Pacurar DI, Perrone I. Adventitious roots and lateral roots: similarities and differences. Annu Rev Plant Biol. 2014;65: 639–66. 10.1146/annurev-arplant-050213-035645 24555710

[43] Bailey-Serres J, Fukao T, Gibbs DJ, Holdsworth MJ, Lee SC, Licausi F, et al Making sense of low oxygen sensing. Trends Plant Sci. 2012;17: 129–138. 10.1016/j.tplants.2011.12.004 22280796

[44] Islam MA, Macdonald SE. Ecophysiological adaptations of black spruce (*Picea mariana*) and tamarack (*Larix laricina*) seedlings to flooding. Trees-Struct Funct. 2004;18: 35–42.

[45] Epstein E. Mineral nutrition of plants: principles and perspectives Wiley; 1972.

[46] Ye ZP. A new model for relationship between irradiance and the rate of photosynthesis in *Oryza sativa*. Photosynthetica. 2007;45: 637–640.

[47] Tyree MT, Patino S, Bennink J, Alexander J. Dynamic measurements of root hydraulic conductance using a high-pressure flowmeter in the laboratory and field. J Exp Bot. 1995;46: 83–94.

[48] Lee SH, Calvo-Polanco M, Chung GC, Zwiazek JJ. Role of aquaporins in root water transport of ectomycorrhizal jack pine (*Pinus banksiana*) seedlings exposed to NaCl and fluoride. Plant Cell Environ. 2010;33: 769–80. 10.1111/j.1365-3040.2009.02103.x 20040068

[49] Jensen CR, Luxmoore RJ, Gundy SD Van, Stolzy LH. Root air space measurements by a pycnometer method. Agron J. 1969;61: 474–475.

[50] Schmidt GW, Delaney SK. Stable internal reference genes for normalization of real-time RT-PCR in tobacco (*Nicotiana tabacum*) during development and abiotic stress. Mol Genet Genomics. 2010;283: 233–241. 10.1007/s00438-010-0511-1 20098998

[51] Yang SF, Hoffman NE. Ethylene biosynthesis and its regulation in higher plants. Annu Rev Plant Physiol Plant Mol Biol. 1984;35: 155–189.

[52] Farquhar GD, Sharkey TD. Stomatal conductance and photosynthesis. Annu Rev Plant Physiol. 1982;33: 317–345.

[53] Else MA, Coupland D, Dutton L, Jackson MB. Decreased root hydraulic conductivity reduces leaf water potential, initiates stomatal closure and slows leaf expansion in flooded plants of castor oil (*Ricinus communis*) despite diminished delivery of ABA from the roots to shoots in xylem sap. Physiol Plant. 2001;111: 46–54.

[54] Araki H. Water uptake of soybean (*Glycine max* L. Merr.) during exposure to O2 deficiency and field level CO2 concentration in the root zone. Field Crop Res. 2006;96: 98–105.

[55] Jackson MB, Davies WJ, Else MA. Pressure-flow relationships, xylem solutes and root hydraulic conductance in flooded tomato plants. Ann Bot. 1996;77: 17–24.

[56] Liu J, Equiza MA, Navarro-Rodenas A, Lee SH, Zwiazek JJ. Hydraulic adjustments in aspen (*Populus tremuloides*) seedlings following defoliation involve root and leaf aquaporins. Planta. 2014;240: 553–564. 10.1007/s00425-014-2106-2 24957702

[57] Rodríguez-Gamir J, Intrigliolo DS, Primo-Millo E, Forner-Giner MA. Relationships between xylem anatomy, root hydraulic conductivity, leaf/root ratio and transpiration in citrus trees on different rootstocks. Physiol Plant. 2010;139: 159–169. 10.1111/j.1399-3054.2010.01351.x 20088906

[58] Kozlowski TT. Responses of woody plants to flooding and salinity. Tree Physiol. 1997;17: 490–490.

[59] Else MA, Davies WJ, Malone M, Jackson MB. A negative hydraulic message from oxygen-deficient roots of tomato plants? Plant Physiol. 1995;109: 1017–1024. 12228649 10.1104/pp.109.3.1017 PMC161404

[60] Colmer TD. Long-distance transport of gases in plants: a perspective on internal aeration and radial oxygen loss from roots. Plant Cell Environ. 2003;26: 17–36.

[61] van Dongen JT, Licausi F. Oxygen sensing and signaling. Annu Rev Plant Biol. 2014;66: 345–367. 10.1146/annurev-arplant-043014-114813 25580837

[62] Reeksting BJ, Olivier NA, van den Berg N. Transcriptome responses of an ungrafted Phytophthora root rot tolerant avocado (Persea americana) rootstock to flooding and *Phytophthora cinnamomi*. BMC Plant Biol; 2016;16: 205 10.1186/s12870-016-0893-2 27658453 PMC5034587

[63] Skirycz A, De Bodt S, Obata T, De Clercq I, Claeys H, De Rycke R, et al Developmental stage specificity and the role of mitochondrial metabolism in the response of *Arabidopsis* leaves to prolonged mild osmotic stress. Plant Physiol; 2010; 152: 226–244. 10.1104/pp.109.148965 19906889 PMC2799359

[64] Boursiac Y, Chen S, Luu DT, Sorieul M, Van Den Dries N, Maurel C, et al Early effects of salinity on water transport in *Arabidopsis* roots. Molecular and cellular features of aquaporin expression. Plant Physiol. 2005;139: 790–805. 10.1104/pp.105.065029 16183846 PMC1255996

[65] Aroca R, Amodeo G, Fernández-Illescas S, Herman EM, Chaumont F, Chrispeels MJ. The role of aquaporins and membrane damage in chilling and hydrogen peroxide induced changes in the hydraulic conductance of maize roots. Plant Physiol. 2005;137: 341–353. 10.1104/pp.104.051045 15591439 PMC548864

[66] Muries B, Faize M, Carvajal M, Martínez-Ballesta MDC. Identification and differential induction of the expression of aquaporins by salinity in broccoli plants. Mol Biosyst. 2011;7: 1322–1335. 10.1039/c0mb00285b 21321750

[67] Vidoz ML, Loreti E, Mensuali A, Alpi A, Perata P. Hormonal interplay during adventitious root formation in flooded tomato plants. Plant J. 2010;63: 551–562. 10.1111/j.1365-313X.2010.04262.x 20497380

[68] Voesenek LACJ, Bailey-Serres J. Flooding tolerance: O2 sensing and survival strategies. Curr Opin Plant Biol. 2013;16: 647–653. 10.1016/j.pbi.2013.06.008 23830867

[69] Bradford KJ, Yang SF. Xylem transport of 1-Aminocyclopropane-1-carboxylic acid, an ethylene precursor, in waterlogged tomato plants. Plant Physiol. 1980;65: 322–326. 16661182 10.1104/pp.65.2.322 PMC440319

