# Supplementary material for: Stable expression of aquaporins and hypoxia-responsive genes in adventitious roots are linked to maintaining hydraulic conductance in tobacco (Nicotiana tabacum) exposed to root hypoxia
Source: PLoS One. 2019 Feb 7;14(2):e0212059. doi: 10.1371/journal.pone.0212059 (PMC6366753; doi:10.1371/journal.pone.0212059)
Supplement: S1 Table — (DOCX) [file pone.0212059.s002.docx]

**S1 Table. qRT-PCR primers for tobacco (*Nicotiana tabacum*) genes**

| Genes | Primers | |
| --- | --- | --- |
| *PIP1;1* | Forward | CCAATTACTGGAACTGGCATCA |
|  | Reverse | CCATGCCTGTTTTTTGTTGAAG |
| *PIP1;2* | Forward | TGCTGGTGTTGTGAAGGGATT |
|  | Reverse | AACATTAGCACCACCACCAAGTC |
| *PIP1;3* | Forward | TGGTATGATCTTTGCCCTTGTCT |
|  | Reverse | CAAATGTCACTGCTGGGTTAATG |
| *PIP1;4* | Forward | GCTGGTGTTGTGAAGGGATTC |
|  | Reverse | AACATTAGCACCACCACCAAGTC |
| *PIP2;1* | Forward | TTGGGTGCTGAGATTATTGGAA |
|  | Reverse | ATGGGAGTCACGGGCACTT |
| *EF1-α* | Forward | TGAGATGCACCACGAAGCTC |
|  | Reverse | CCAACATTGTCACCAGGAAGTG |
| *L25* | Forward | CCCCTCACCACAGAGTCTGC |
|  | Reverse | AAGGGTGTTGTTGTCCTCAATCTT |
| *ADH1* | Forward | AAGCTGGAGGAATTGTGGAGAG |
|  | Reverse | ACCAGTGAACACAGGAAGAACA |
| *PDC1* | Forward | CCCGAACACCATTCACATTTC |
|  | Reverse | CATCAGCAGATTCCACGATTTC |
| *ACS* | Forward | AAACGAGCCATTGCAACAAAGA |
|  | Reverse | TGAATCCTGGTAAGCCCATGTC |
